# Therapeutic ultrasound for glaucoma: clinical use of a low-frequency low-power ultrasound device for lowering intraocular pressure

**DOI:** 10.1186/2050-5736-2-15

**Published:** 2014-09-26

**Authors:** Donald Schwartz, John Samples, Olga Korosteleva

**Affiliations:** 1 Long Beach Eye Care Associates, 2650 Elm Avenue #108, Long Beach, CA 90806, USA; 2 USC Eye Institute, Los Angeles, CA, USA; 3 UC Irvine Gavin Herbert Eye Institute, Irvine, CA, USA; 4 Rocky Vista University, Parker, CO, USA; 5 Department of Mathematics and Statistics, California State University, Long Beach, CA, USA

**Keywords:** Ultrasound, Glaucoma, Trabecular meshwork

## Abstract

**Background:**

This is a first-in-human study to determine the efficacy and tolerability of a new method of treating glaucoma using a low-power, low-frequency, focused therapeutic ultrasound for glaucoma (TUG) device designed to trigger an inflammatory reaction in the anterior chamber angle and trabecular meshwork to enhance outflow. The use of the device is anticipated for mild or moderate open-angle glaucoma as an enhancement to outflow.

**Methods:**

In a two-branch clinical trial, a total of 26 primary open-angle glaucoma patients underwent a procedure consisting of the external application of the TUG device. In branch 1, nine of these patients were naïve to pharmaceutical treatment or had been off of medication for over 6 months. In branch 2, 17 patients were treated after a medication washout period. All patients in the study were followed for 12 months.

**Results:**

In branch 1, there was a decrease in intraocular pressure averaging over 20% lasting at least a year in 74% of the eyes with non-normotensive open-angle glaucoma. In branch 2, an average of two visits while on medication provided the comparison intraocular pressure (IOP) to the effect of the TUG treatment after washout. It was seen that the intraocular pressure over the year post-treatment was equal to or better than the pharmaceutical control in close to 80% of measurements.

**Conclusion:**

A novel device for lowering intraocular pressure is described with a potential for adding to our armamentarium for treating glaucoma. This is a small cohort study which indicates beneficial trends.

**Trial registration number:**

The study was a registered clinical trial, #ISRCTN50904302.

## Background

Open-angle glaucoma is a worldwide problem for which newer, portable, low-cost, and effective treatments are needed. Glaucoma affects approximately 3 million people in the United States (source: preventblindness.org—Prevent Blindness America) and 70 million worldwide (source: glaucoma.org—Glaucoma Research Foundation). It is expected that the increasing age of our population will significantly increase the number of people with this blinding disease. The present methods used to treat glaucoma have significant drawbacks. Pharmaceutical agents must depend on compliance, often have side effects, and may interfere with other medications required by the patient. The use of lasers such as argon or selective laser trabeculoplasty offers a useful alternative to many of the problems inherent with pharmaceutical agents. However, these instruments require a slit lamp biomicroscope apparatus for viewing the trabecular meshwork and a contact lens system for application of the energy for such treatment.

Cataract surgery lowers intraocular pressure in patients with coexisting glaucoma [1-6]. The basis for this effect may be due to both anatomic and biochemical changes in the area of the trabecular meshwork since observations have suggested that the use of ultrasound in the eye may result in a decrease in pressure [7]. The association of the decrease in pressure with cataract surgery became more evident when the use of phacoemulsification (ultrasound) became prevalent. The finding that there is a decrease in the intraocular pressure (IOP) after phacoemulsification has often been attributed to an increase in the opening of the angle. This is certainly true with narrow-angle glaucoma, but recent studies have revealed no correlation between the change in chamber depth and the IOP decrease with open-angle glaucoma whereas the decrease is correlated with the pre-treatment IOP [8-11]. In addition, reports of cataract surgery performed with an intracapsular lens extraction did not indicate a decrease in IOP. Intracapsular cataract surgery did not involve the use of ultrasound. Radius et al. reported that after the intracapsular cataract surgery, there was a slight increase in the IOP [12].

**Figure 1:**
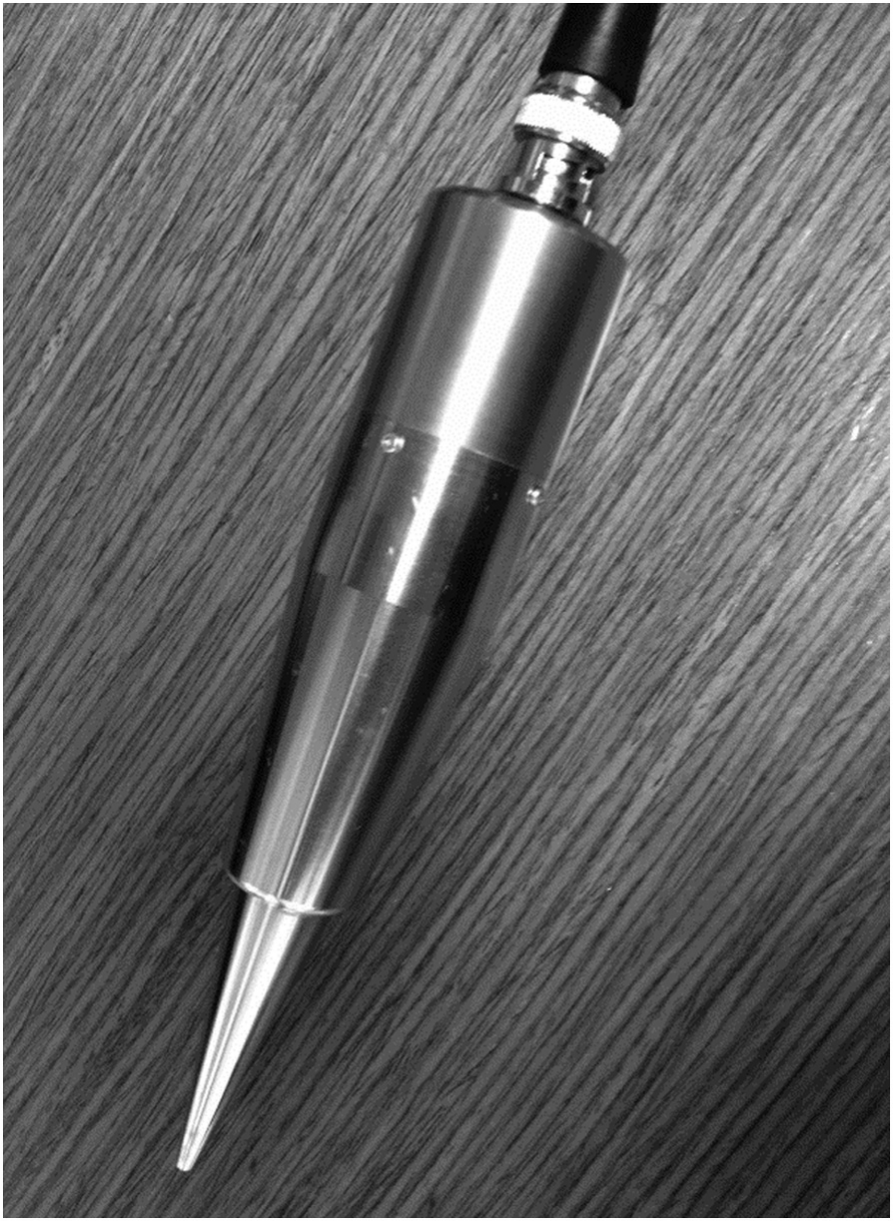
A prototype TUG instrument hand piece.

A prototype instrument hand piece (see Figure 1) was designed and built to produce low-frequency ultrasound of 40 kHz. This frequency is the same as that of a typical cataract surgery ultrasound. Although the frequency is the same, the energy from the bubbles created by phacoemulsification can create cavitation with a temperature of over 7,000°C; the therapeutic ultrasound for glaucoma (TUG) treatment power of less than 2 W/cm^2^ only allows a temperature within the focal area to reach 45°C [13,14]. The instrument was developed for the external application of ultrasound with the purpose of decreasing the intraocular pressure. We hypothesized that the application of low-power and low-frequency focused ultrasound energy to the trabecular meshwork could result in the lowering of intraocular pressure by triggering a similar cytokine cascade to that triggered by SLT laser. To verify the hypothesis, we designed a prospective study.

## Methods

The author confirms that (1) the research followed the tenets of the Declaration of Helsinki, (2) informed consent was obtained, (3) the research was approved by the institutional review board to allow clinical studies (on the basis of previous animal studies supporting a non-significant risk classification), and (4) power analysis was performed to justify the number of patients enrolled in the study.

### Device development

Prior to the use of the instrument, a power determination was performed with *in vitro* and *in vivo* animal studies based on a temperature elevation of approximately 6°C. This temperature elevation was just below that which causes pain and cell necrosis [15-17]. It was found that with external application of the TUG device of approximately 3 W of power/cm^2^ to the hand piece, the temperature increased and then stabilized at approximately 43°C, when measured by a “K type” microthermocouple positioned 0.5 mm below the surface of a pig eye at the limbus using an Omega HH508 digital thermometer. The instrument was developed and tested with this microthermocouple to determine the power required to raise the temperature by 7° from baseline. Testing was then repeated with pig eyes raised to a basal temperature of 36.5°C by water bath to determine the power to raise the temperature to 43°C to 44°C and maintain this steady-state increased temperature.

*In vivo* work on the animal model at power settings above 4 W/cm^2^ resulted in a large corneal inflammatory reaction. Further *in vivo* studies therefore used only power settings below this level. A point was then chosen on the sclera side with 0.5 mm of clearance from the limbus for this study.

### Study description

In early clinical work, the first two series TUG.1 and TUG.2 (not published) were conducted to determine the tolerability (TUG.1) and to refine the parameters (TUG.2) for treatment. The presently reported study (TUG.3) was a prospective controlled study using the ultrasound instrument in the manner which optimized the intraocular pressure-lowering effect.

This study was funded by EyeSonix Incorporated and followed a protocol approved by Western Institutional Review Board (WIRB). The study was conducted at one site in the Long Beach Eye Care Associates offices. Health Insurance Portability and Accountability Act (HIPPA)-compliant informed consent was approved by WIRB. Each patient who participated in the study (or a designated family member) gave written consent. The study was a registered clinical trial, #ISRCTN50904302.

### Patient selection

Recruitment was conducted among glaucoma patients seen at the study site.

The eligibility criteria were twofold:

1. Patients with open-angle glaucoma and without medical or laser treatment for at least 6 months, or

2. Patients presently on pharmaceutical treatment for glaucoma.

The exclusion criteria included both previous invasive glaucoma surgery and an inability to comply with follow-up visits. Treatment-naïve patients were offered the option of pharmaceutical agents or laser (always in this order) and also the option of being part of the study.

### Randomization and treatment protocol

Patients with symmetric IOP were randomized by a coin flip as to the eye selected for the TUG treatment. If there was a significantly higher IOP in one eye, this eye was selected for the TUG treatment.

**Figure 2:**
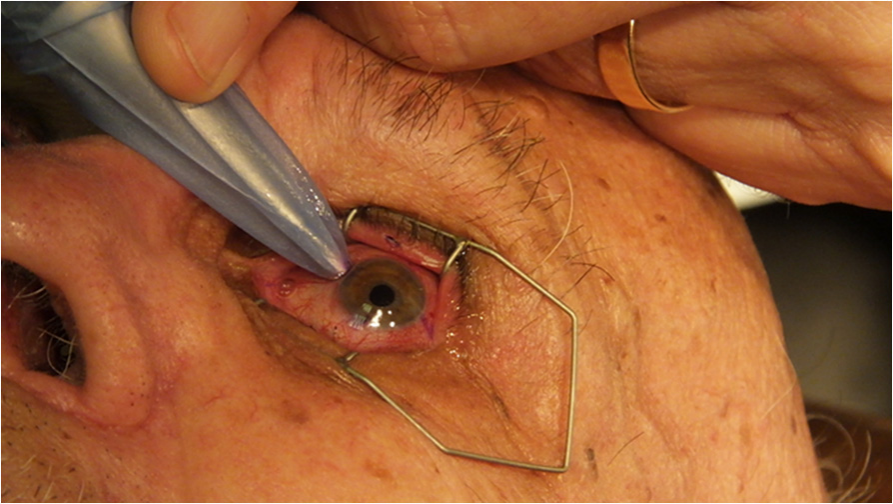
The investigator is performing a glaucoma procedure with the TUG device.

If patients were presently on pharmaceutical agents, a bilateral washout period was performed. Prostaglandin analog medications were washed out for 1 month. Other agents were washed out for 1 week.The treatment was performed after a measurement of the IOP was taken, and an ocular examination was conducted to determine the baseline IOP. The baseline IOP was an average of the two IOP measurements prior to the TUG treatment. In addition, an evaluation was performed to characterize any baseline signs of inflammation. The following steps were performed during the surgery illustrated in Figure 2. A drop of tetracaine was applied. The patient was then placed in the supine position. A lid speculum was used for exposure. A marking pen was then used on the sclera to mark four equally spaced quadrants at the limbus. The eye was then covered with lidocaine 3.5% gel (Akorn). This served as the necessary transmission gel, an added anesthetic. Additionally, it maintained corneal moisture. A function generator (Tektronix AFG3101 Single Channel Arbitrary/Function Generator, calibrated in October of 2008) was then tuned to generate the proper power and frequency. This was fed by the single-channel output to a power amplifier (E&J RF Power Amplifier, Model #2100 L, Serial #1045). The “EyeSonix” hand piece of 40 kHz was then attached. The treatment was performed once the correct power output with zero reflectance was determined by tuning the frequency.

A tip cover was then put over the hand piece. Next, the tip was placed sequentially at the treatment sites. The application was at a point 0.5 mm on the scleral side of the limbus. Applications were 45 s in duration at each clock hour position for 12 clock hours. The four previously placed marks allowed for an equal positioning of the spots of three per quadrant. Throughout the procedure, the power output was monitored and adjusted to maintain the correct delivery of energy to the hand piece. The intraocular pressure was measured 1 h after the treatment as a safety precaution, since after laser trabeculoplasty there is a small chance of a pressure spike after the treatment. No significant post-treatment pressure elevations were seen. Post-treatment, the patients received Nevanac (nepafenac ophthalmic suspension) 0.1% three times a day for 1 day. This non-steroidal medication was used if there were symptoms of inflammatory response: soreness, photophobia, or signs of inflammation; the treatment continued until the symptoms and signs resolved. Steroids, although more powerful, were avoided as the post-inflammatory response was desired. The patients were seen 14 times post-surgically: on the following day, 1 week later, and after that once a month for 12 months. The measurements of IOP were not taken in 17 patients for a total of 47 missed follow-up visits to the investigator’s office. Two patients were lost to the follow-up visits after the 6-month appointment, and two dropped out after being in the study for 10 months.

Results of the treatment were tabulated at each follow-up visit into two general categories. The first category was that of the intraocular pressure. The pre-treatment (or baseline) IOP reading was an average of the two most recent readings prior to the washout. These were Goldmann applanation measurements. Post-treatment readings of both eyes were obtained by the technician before the investigator entered the room. Post-treatment tonometry was performed in two different methods. “Tonopen” (Tono-Pen XL Medtronic) measurements were taken by well-trained technicians with at least four separate readings. These readings were then averaged. After the tonopen measurements were recorded, the technician performed Goldmann applanation tonometry. A second Goldmann applanation tonometry was performed by the investigator. The two Goldman readings were then averaged with the average of the tonopen readings; therefore, the Goldman readings were weighted at 66% of the average for the single overall reported average. The Goldmann tonometer was calibrated at least once a week.

The second category was of an analysis of the inflammatory reaction from the treatment. This evaluation included the subjective symptoms of irritation, discomfort, and pain and an objective slit lamp evaluation for signs of conjunctival injection and signs of anterior chamber cells and flare. Each of the parameters was graded on a 0 to 4 scale (minimal to maximal presence). The subjective symptoms were elicited by staff technicians and verified by the investigator and were always asked in the same manner. The slit lamp evaluation was performed by the investigator. Each of the markers was characterized on a scale of 0 to 4 with 0 being minimally present and 4 the worst possible. These subjective responses were obtained by assistants and verified by the investigator. The signs were observed at the same slit lamp at each visit. The conjunctival injection was observed under low magnification in a low-light room condition whereas cell and flare were evaluated with high power in a dark room.

## Results

### Intraocular pressure

The IOP was analyzed separately in medication-free patients, in medication-free non-normotensive glaucoma patients, and in those who were previously on medication.

#### Medication-free patients

**Figure 3:**
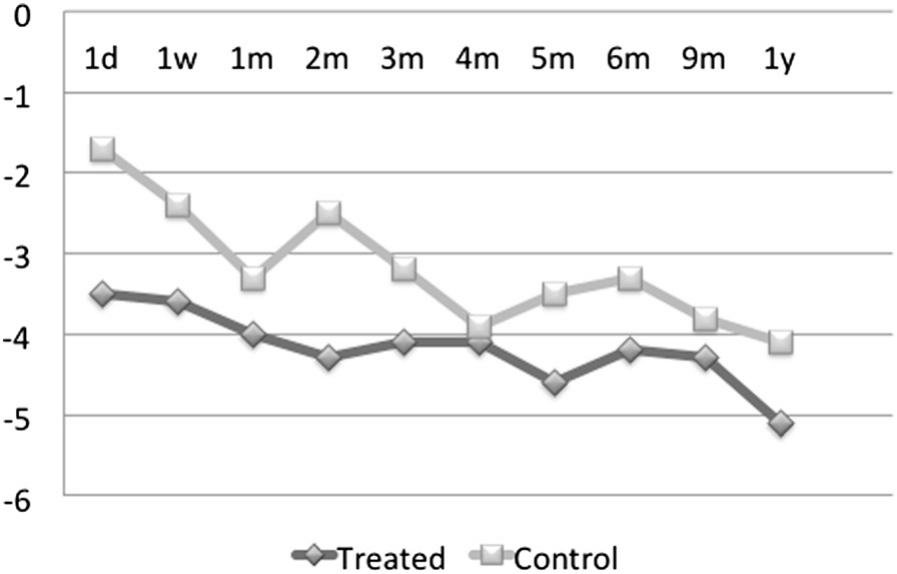
**Average IOP decrease (in mmHg) from the baseline in medication-free patients in treated v. control eyes, *N*** **= 9.**

**Figure 4:**
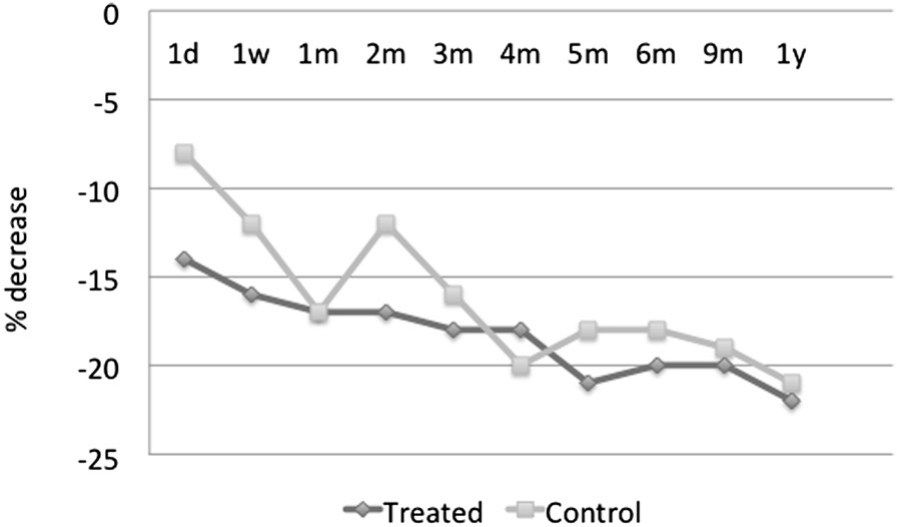
**Average percent IOP decrease from the baseline in medication-free patients in treated v. control eyes, *N*** **= 9.**

For the nine patients, the mean age was 73.3 ± 9.0 years (min = 62 years, max = 89 years). Eight patients were Caucasian and one was African-American. One patient was female and eight patients were male. Two patients had primary open-angle glaucoma, four had normotensive glaucoma, and one had pigmentary glaucoma. In Figure 3, the graph of an average IOP decrease from the baseline for the treatment and control groups is presented. In both groups, there is an overall downward trend in average IOP, with the control group profile remaining above but shadowing that of the treatment group.The average percent decrease in the IOP from the baseline value is represented in Figure 4. The trend line from the time of treatment over the 12-month period reveals a decrease in pressure in both the treated and the contralateral eye which persists throughout the duration of follow-up. Note that for these medication-free patients, the average percent IOP reduction from the baseline is over 20% 1 year post-treatment in either eye.

#### Medication-free non-normotensive glaucoma patients

When reviewing the findings, it became apparent that the decrease in intraocular pressure was less evident with those whose pre-treatment pressure was below 19.5 mmHg. Thus, the analysis was redone without the four normotensive glaucoma patients in order to obtain further perspective in considering the treatment on patients with intraocular pressures of 20 or greater.

**Figure 5:**
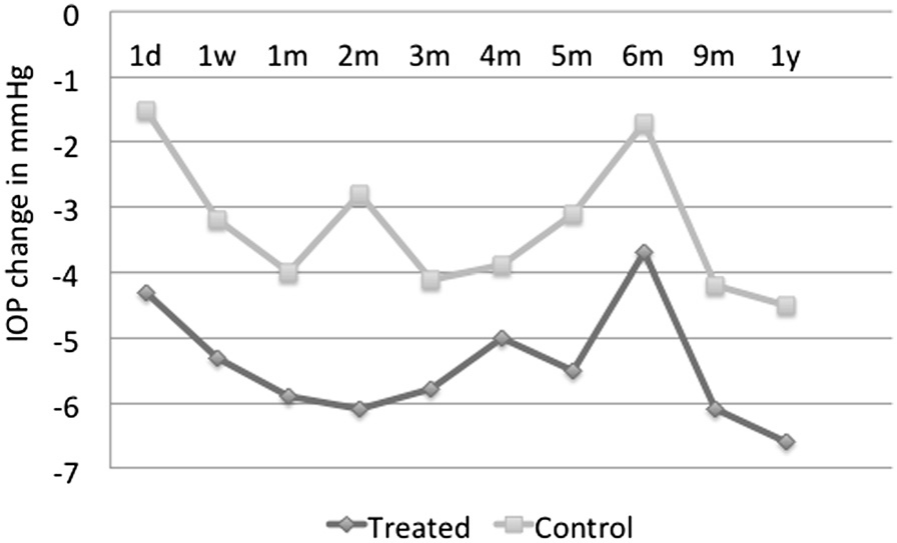
**Average IOP decrease (in mmHg) from the baseline in medication-free non-normotensive glaucoma patients in treated v. control eyes, *N*** **= 5.**

**Figure 6:**
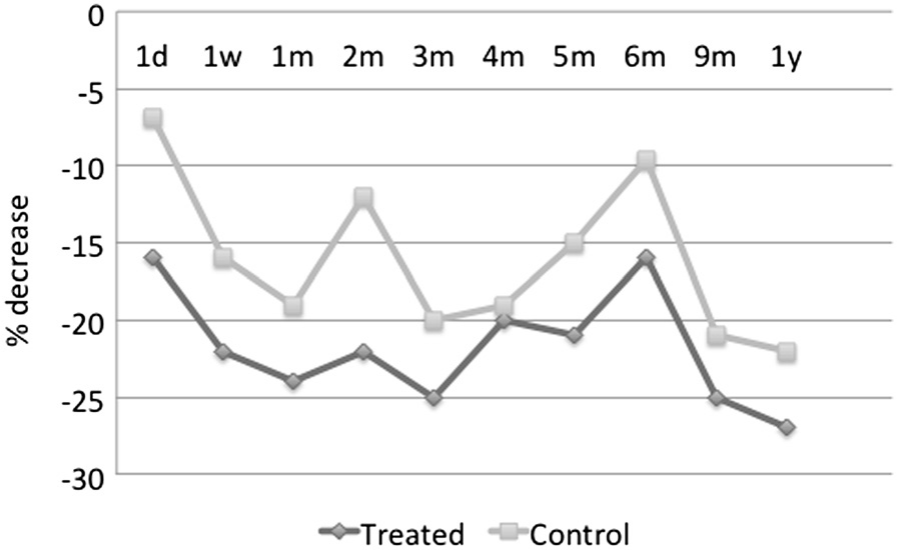
**Average percent IOP decrease from the baseline in medication-free non-normotensive glaucoma patients in treated v. control eyes, *N*** **= 5.**

For the remaining five patients, the mean age was 71.8 ± 11.8 years (min = 62 years, max = 89 years). All patients were Caucasian males. Four patients had primary open-angle glaucoma and one had pigmentary glaucoma. Of these five patients, one missed two follow-up visits, and thus, the data were unavailable in the two cases.Figures 5 and 6 show, respectively, the average IOP decrease and average percent IOP decrease from the baseline for this group of patients. On both graphs, larger decreases in absolute units as well as in percent intraocular pressure are observed in the medication-free patient group (cf. Figures 3 and 4). The average percent IOP decrease from the pre-treatment value approaches 25% as opposed to 20% for all medication-free patients.

#### Example of a medication-free non-normotensive glaucoma patient

**Figure 7:**
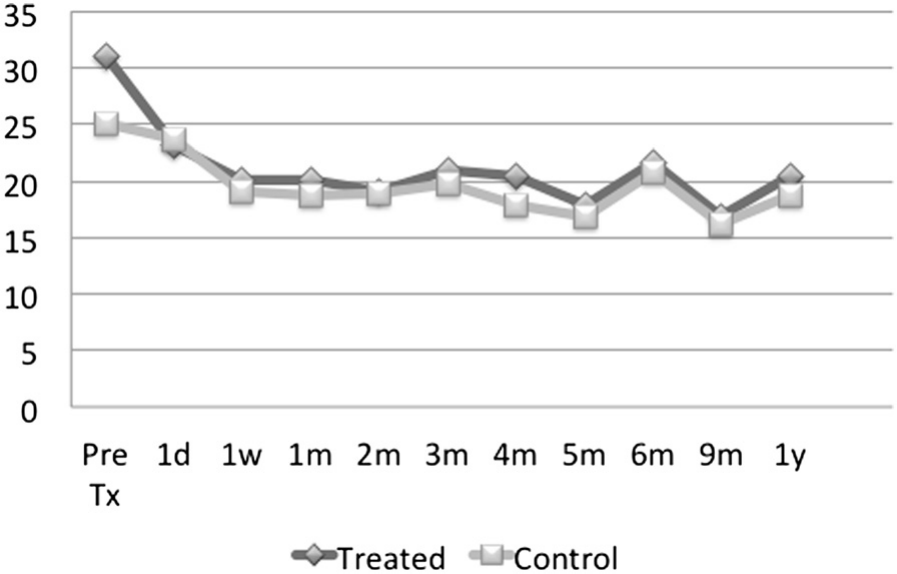
Individual profile for a medication-free non-normotensive glaucoma patient: IOP measurements in treated v. control eyes prior to the TUG treatment and during the study follow-up.

Figure 7 presents an IOP profile for a non-normotensive glaucoma study participant. This person had been a patient in our practice for 3 years with normal pressures and cup disc ratios. He was seen for routine examination in 2010 and found to have increased optic nerve head cupping and an elevated IOP of 32 mmHg by Goldmann applanation tonometry. He was then given options for treatment and chose the TUG study. The IOP measurements were taken at pre-treatment, 1 day and 1 week post-surgery, and then every month for 1 year. His central corneal thickness (CCT) measurements were 526 and 530 μm in the treated and the control eye, respectively.

Note that the control eye exhibits a similar behavior in terms of the IOP measurements to the treated eye. Noteworthy is the length of this effect in both eyes after only one treatment in one eye for this patient.

#### Medication washout patients

The mean age of the 17 patients was 73.1 ± 13.2 years (min = 51 years, max = 92 years). Fourteen patients were Caucasian, one African-American, and one Hispanic. Six females and 11 males were treated. Eight patients had primary open-angle glaucoma, seven had normotensive glaucoma, and two had pigmentary glaucoma. Twelve patients missed a follow-up, but came back for subsequent visits. Two patients dropped out after being in the study for 6 months, and two dropped out after 10 months.

In this group of 17 medication washout patients, three patients had a second TUG treatment within 1 year. Such retreatments were performed if the intraocular pressure approached the washout pressure. These retreatments occurred at either the fifth or the sixth month post-initial TUG treatment. Also in this group of patients, four of the 17 had a reintroduction of medication. It should be noted that the washout of the medication was for both eyes. One of the patients had a resumption of medication in the control eye, but not in the treated eye. Of the group that went back on medication, one patient used the medication for only 1 month and found the medication again led to unacceptable irritation. She then was one of the four who had a second TUG treatment.

**Figure 8:**
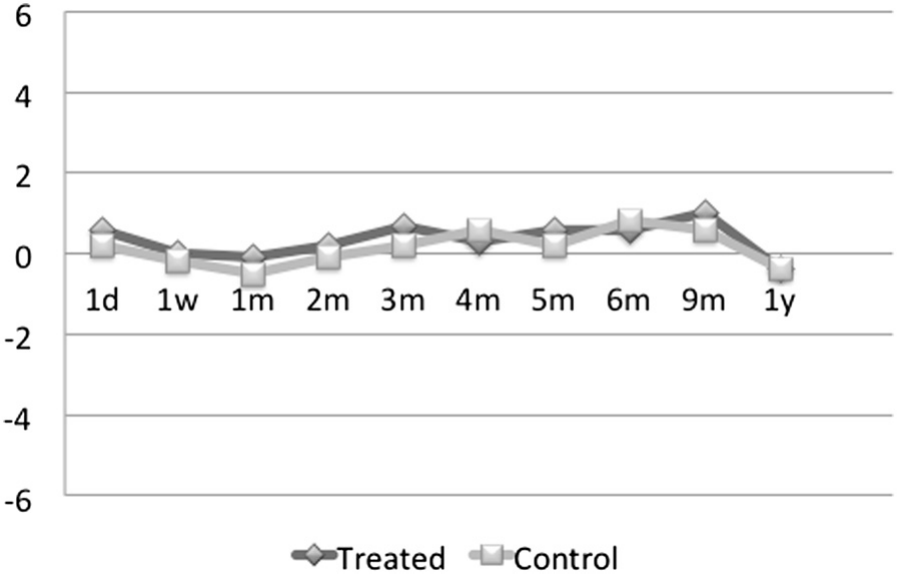
**Average IOP difference from pharmaceutical control in washout patients in treated v. control eyes, *N*** **= 17.**

**Figure 9:**
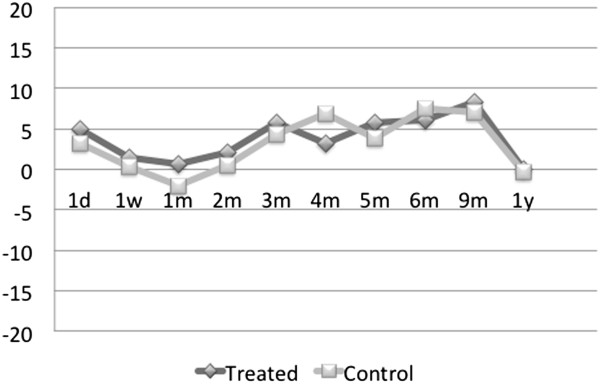
Average percentage change in IOP from the pharmaceutical regimen.

**Figure 10:**
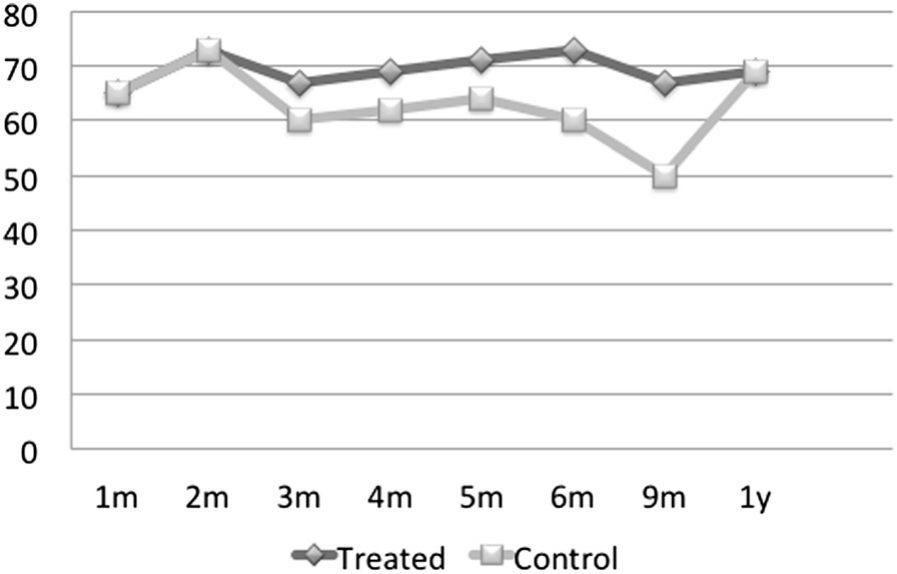
**Percent of patients in the medication washout group (*N*** **= 17) whose IOP change from the baseline value was within 10% or below the pharmaceutical control: treated v. control eyes, *N*** **= 17.**

The baseline IOP value for these patients was an average of the IOP measurements for two visits prior to the medication washout period. The effect of the TUG treatment in the IOP reduction was compared to the IOP on the pharmaceutical regimen.Figure 8 shows the average change in IOP from the baseline after the initial TUG treatment. Where the graph is above zero, the post-TUG treatment IOP was higher, below zero—the medication IOP was higher. Note that the two IOP measurements deviate very little from each other always remaining within ±2 mmHg.Figure 9 represents the same results as in Figure 8 but in terms of the average percent of change in IOP from the patient’s pharmaceutical regimen. The positive values on the graph indicate that the post-TUG treatment IOP exceeds that of the pharmaceutical regimen.To analyze the number of patients who retained clinical control of pressure after washout, a connected graph of percent of medication washout patients with IOP change from baseline of at most 10% is constructed (see Figure 10).

#### Examples of medication washout patients

**Figure 11:**
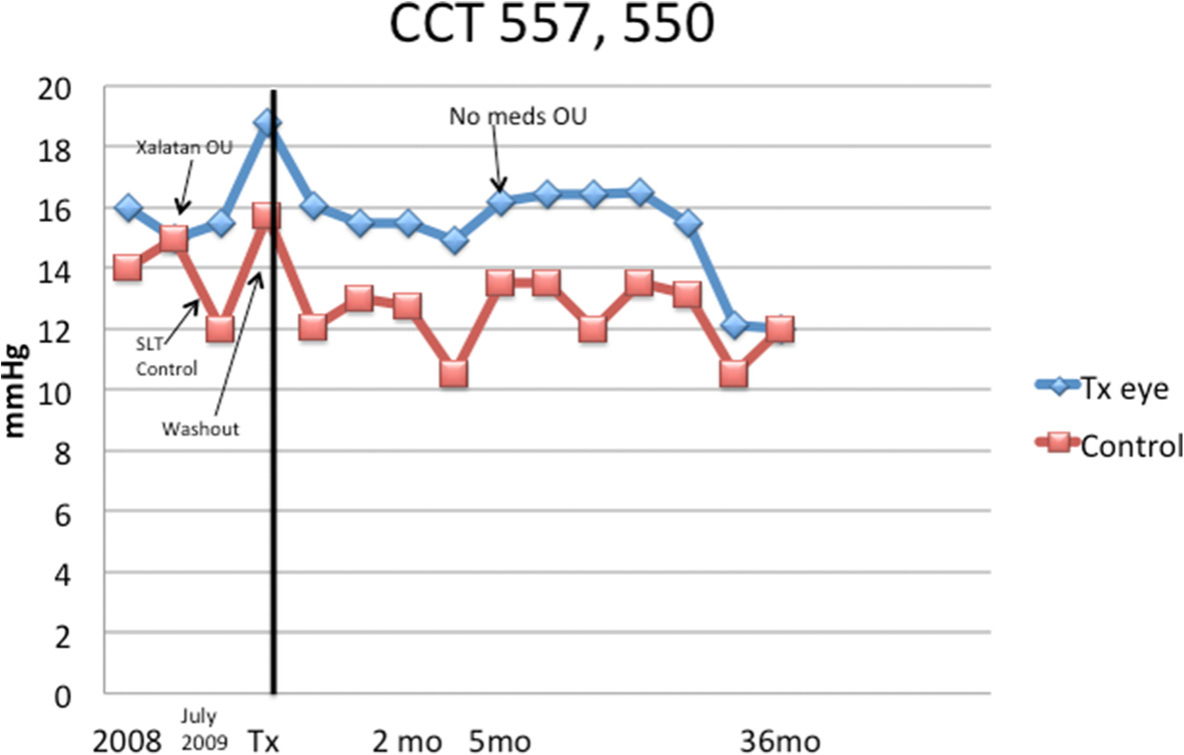
Historical perspective for a medication washout patient: IOP measurements in both eyes prior to the TUG treatment and during the study follow-up.

**Figure 12:**
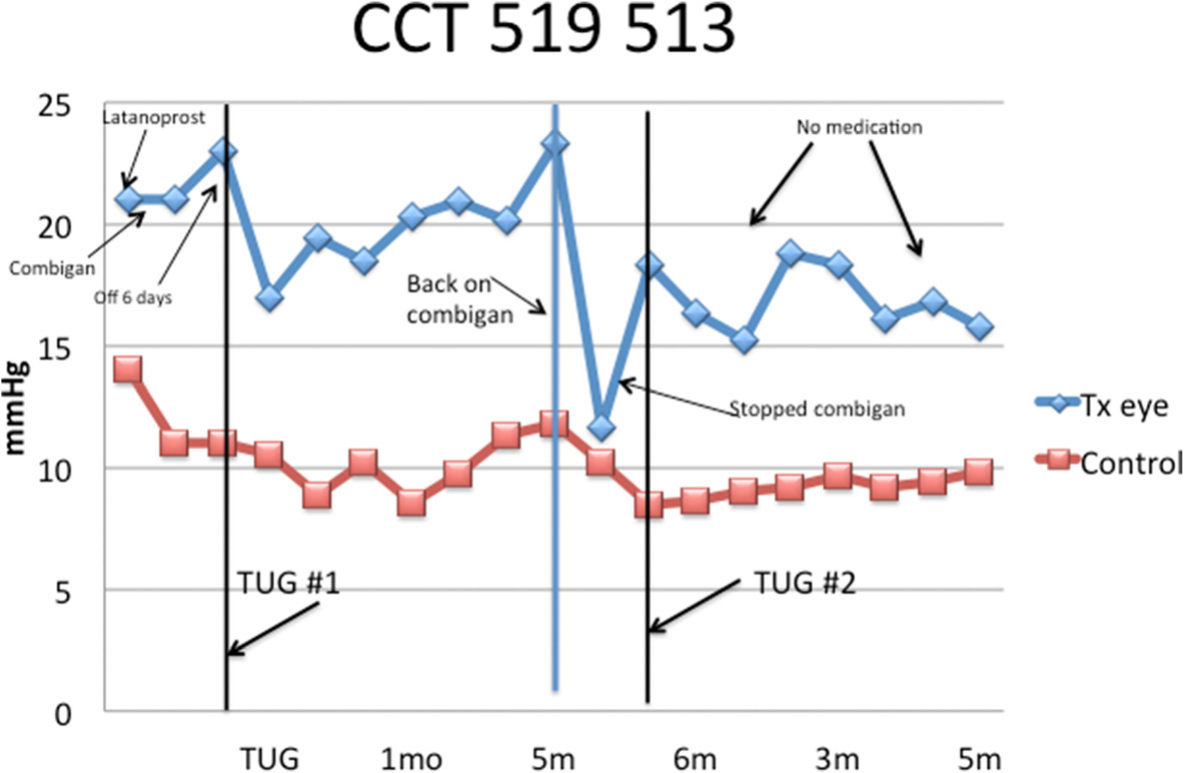
Historical perspective for a medication washout patient who resumed medication and underwent a second TUG treatment: IOP measurements in both eyes prior to the first TUG treatment and during the study follow-up.

**Figure 13:**
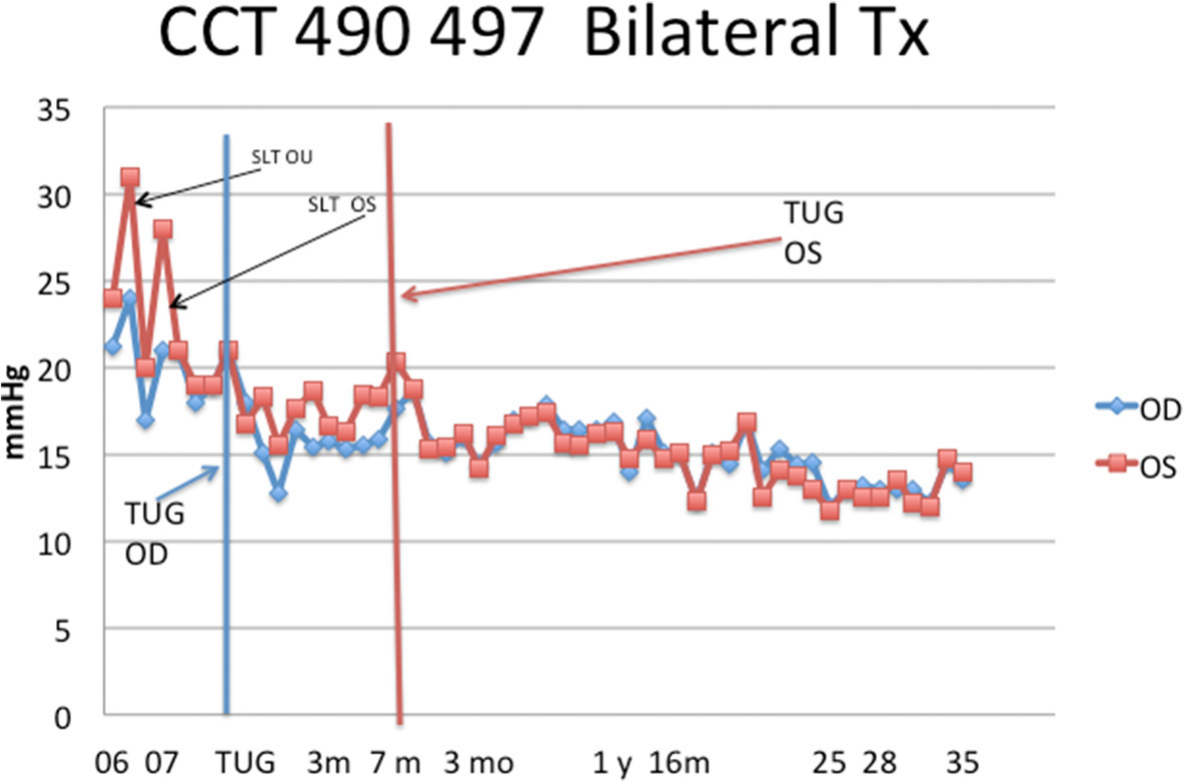
**The first subject to undergo the TUG treatment in each eye.** The second eye was treated 7 months after the first eye when the intraocular pressure in that eye had increased to the original baseline.

Figure 11 demonstrates an historical perspective of IOP measurements in both eyes across the visits for a patient in the medication washout group. This patient was among the 13 (74%) patients who needed only a single TUG treatment to last 1 year. In the past, he had been on latanoprost which controlled his IOP to approximately 16. He had a washout of this medication. At the end of the 1-year period, he was off medication in both eyes with an IOP around 16 mmHg. Even longer follow-up shows his IOP to be maintained at a significant decrease from baseline and even from pharmaceutical control.The next graph (Figure 12) illustrates an IOP historical perspective for a patient who was compelled to return to medication with additive effect and later chose to have a TUG retreatment which showed an enhanced effect from the second treatment over the original. This patient was on latanoprost and then changed to Combigan as a result of side effects of the latanoprost. Her IOP on medication was 21 mmHg. She had a 1-week washout of the Combigan with a resultant IOP of 23 mmHg. Her TUG treatment led to a decrease in IOP to 17 mmHg, but it gradually increased to 23 mmHg over a 5-month period of time. She was offered the option of returning to medication. She was reintroduced to medication with a substantial decrease to 12.5 mmHg. But she again was bothered by the side effects of Combigan and elected to have a second TUG treatment after washout. The second TUG treatment was performed, and after 7 months post-TUG #2 (1 year after TUG#1), the IOP was 15 mmHg without medication.The first subject to have bilateral treatment is illustrated (Figure 13). His pressure gradually elevated over time after SLT laser. He elected to have TUG rather than a repeat SLT or initiation of pharmaceutical agents. After coin flip, the first treated was the right eye (OD). There was a profound effect on this treated eye. The non-treated eye appeared to have a modest effect. After 7 months, the non-treated eye returned to baseline. At this time, the left eye (OS) was treated. The graph shows that for close to 3 years after the treatment of the second eye, the intraocular pressures are significantly lower than baseline in both eyes.

### Tolerability of the procedure

The intraocular pressure was measured at a time approximately 2 h after the treatment to look for any possible pressure spikes. No pressure spikes were found in the patients treated.

The tolerability of the treatment with the TUG device was evaluated at each visit. Patients were queried about three markers of patient symptoms: irritation, discomfort, and pain. Similarly, there were three findings which we associated with signs of inflammatory response: conjunctival injection, anterior chamber cells, and flare.

The values of symptoms scores were as follows:

1. *Irritation* scores ranged between 1 and 2.5 in three patients at the 1-day visit. One week after the treatment, irritation was gone.

2. *Discomfort* was present in four patients at the 1-day visit. One had a score of 2, and three were at 1. At 1 week post-treatment, one patient was at 1, one at 1.5, and the rest were at 0. Afterwards, this symptom has disappeared.

3. *Pain* was experienced by one patient at the level of 2 at the 1-day visit. Besides this instance, pain was absent for each and every patient at each and every visit.

The values of signs scores are listed below:

1. *Injection of the conjunctiva* was a frequent but short-lived finding. At the first visit, 1 day after the treatment, it was found in 24 of the 26 patients. One had a score of 3, one had a score of 2.5, and seven were at 2. There were five at 1.5, seven at 1, and the other three scored 0.5. By the 1-week visit, only eight had any such finding: two at 0.5 and six at 1. This sign was not apparent afterwards.

2. *Cells* in the aqueous humor were not observed in any of the patients at any visit.

3. *Flare* was observed in the treated eye of nine patients on the first day. In five patients, the score was 0.5, and the other four had a score of 1. At the 1-week visit, a flare score of 0.5 was measured in a single patient. In the other patients, this sign was absent.

**Figure 14:**
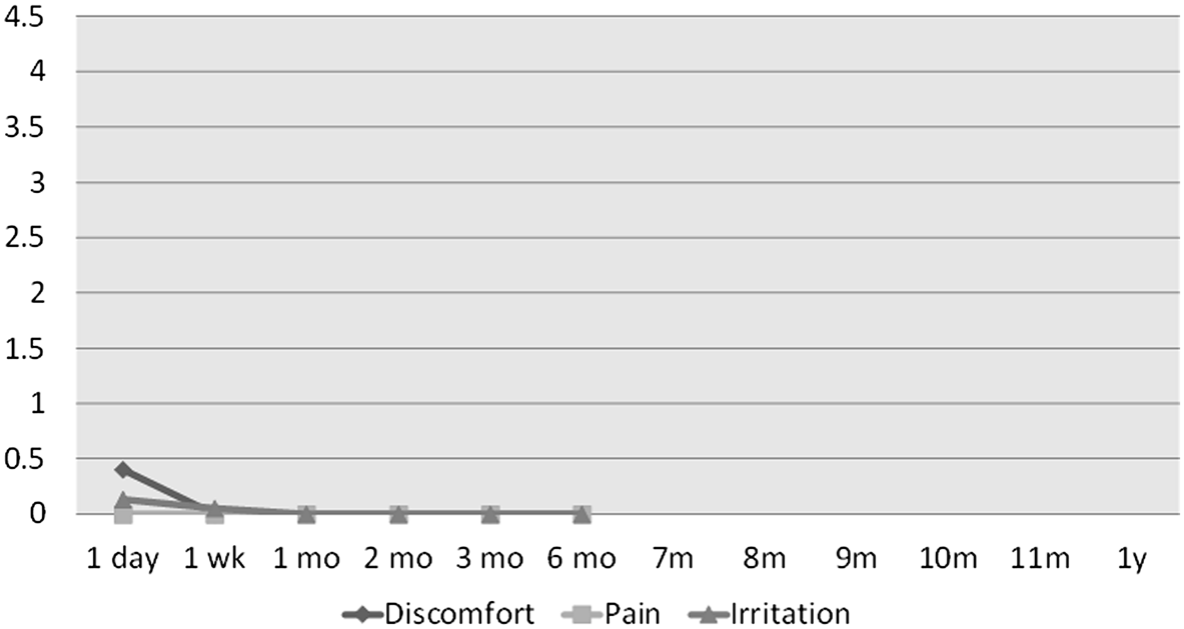
Average symptoms scores (irritation, discomfort, and pain) in TUG patients over the duration of the study.

**Figure 15:**
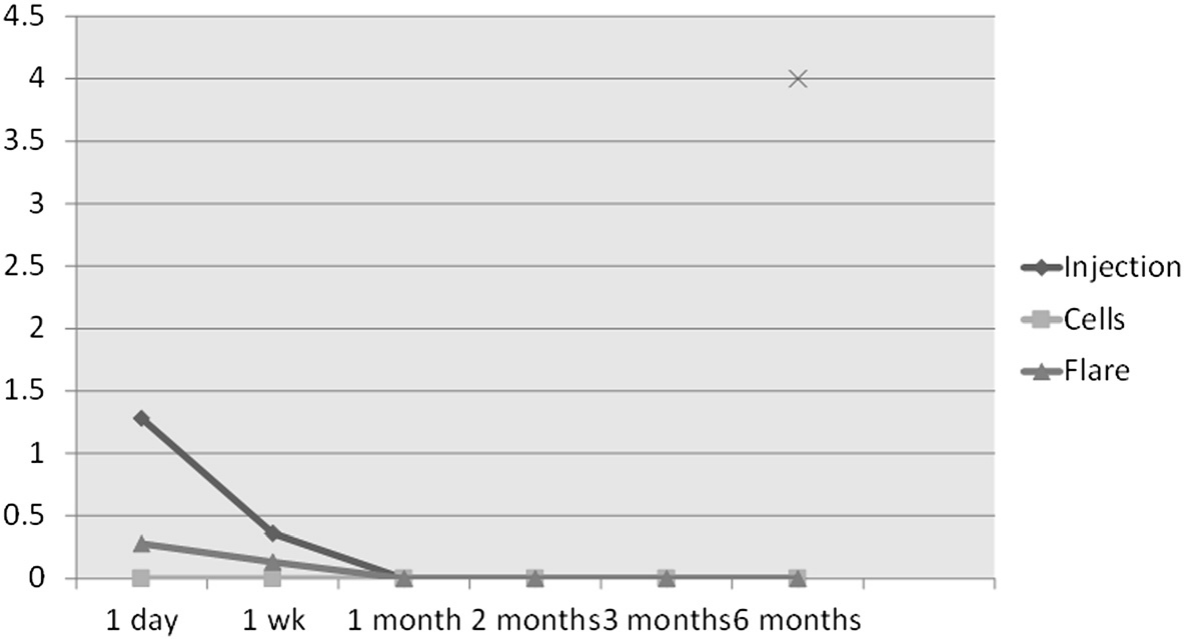
Average signs scores (injection, cells, and flare) in TUG patients over the duration of the study.

In summary, there is a frequent finding of conjunctival injection with a subjective feeling of irritation that fades over several days. No symptom or sign reached the level of 3 at any time. The more serious symptom of pain and sign of cells in the aqueous humor were remarkable for their absence at each and every visit including the first day (with one exception of pain on day 1).The average symptoms and signs scores are shown in Figures 14 and 15, respectively. As can be seen on the graphs, the minimal findings have dissipated by 1 month. It is doubtful that new signs or symptoms will appear in the longer follow-up.

From the graphs, the patients tolerated the procedure quite comfortably with the only common comment of mild irritation which was consistent with a mild to moderate conjunctival injection. These typically resolved in a few days in almost all cases without treatment. If the patient had these findings, Nevanac (nepafenac ophthalmic suspension) was offered. Most patients declined to use the medication and stated that the discomfort did not warrant any pharmaceutical treatment.

## Discussion

The armamentarium of treatment modalities for glaucoma is increasing. At the present time, pharmaceutical agents are typically the first line of treatment with the use of laser seen as an adjunct method. A new method of decreasing intraocular pressure is described. This is the initial study to be reported using a low-frequency, low-power, focused ultrasound for the treatment of glaucoma.

The mechanism of action has yet to be described fully, but the design of the ultrasound was with the purpose of creating a focal area of hyperthermia within the anterior chamber angle. It was felt that such an effect could trigger inflammatory cytokines analogous to the effect of SLT laser. The finding of a concurrent decrease in the intraocular pressure in the contralateral eye may support this mechanism of action.

We hypothesized that ultrasound energy applied externally near the limbus may have at least three modes of action all of which could trigger a decrease in the intraocular pressure. None of these potential modes of action are mutually exclusive, and other modes are certainly possible.

Firstly, there may be a sonomechanical, or vibratory, effect transmitted to the trabecular meshwork, loosening debris and flushing out blockages [18-24]. Work in Sweden by Björn Svedbergh resulted in a patented device with the expressed concept of shaking debris to loosen trabecular meshwork blockage (Ultrasound Probe—US Patent 6162193, filing date Sep 15, 1997, issue date Dec 19, 2000). This device utilized a fluid-filled chamber with a membrane for applying non-focused ultrasound transmission to the external eye.

A second mechanism, that of a localized hyperthermia, may trigger heat shock proteins and potentially beneficial cytokines. This would hypothetically be similar to a cytokine response evoked by laser trabeculoplasty [25-33]. Specific cytokines have been demonstrated to lower the IOP after both argon and selective laser trabeculoplasty and have also been found to be triggered by ultrasound of a frequency similar to that used in phacoemulsification [34].

A third mode of effect may be an induction of cytokines through integrins [35,36]. These receptors absorb ultrasound energy and in turn induce cytokine activity which may be beneficial in lowering the IOP.

Ultrasound energy applied externally to the eye has a significant advantage compared with many other treatment modalities in that it can be applied in a non-invasive manner. Previous treatments to use ultrasound to treat glaucoma have been directed more posteriorly [37-42]. Coleman et al. revealed that high-intensity focused ultrasound (HIFU) led to an ablation of the ciliary body and a subsequent thinning of the sclera. The energy was directed from the outside of the eye towards the ciliary body with an attempt to ablate the tissue and thereby decrease aqueous production. This high-powered ultrasound had an additional effect in leading to a thinning of the sclera overlying the ciliary body. The use of this ultrasound model was for intransigent glaucoma, and its use fell from favor with the advent of other superior treatment methods. Recently, another group (EyeTechCare of Lyon, France) has reported an ultrasound device, “EyeOP1”, used to coagulate the ciliary epithelium. This device uses a single-treatment strategy with a circular tip with a multiple port array. These ports focus high-intensity ultrasound into the ciliary body to decrease aqueous inflow in patients with refractory glaucoma [17].

In a single-center pilot study of a novel glaucoma treatment technique (TUG), we found a significant decrease in intraocular pressure. The effect on intraocular pressure in the “naïve” group was significant with an average decrease in the total group of almost 20% and almost 25% when not including the normotensive glaucoma patients. The effect of the treatment appeared to be more significant in the higher baseline intraocular pressures. In over 74%, the effect lasted for 1 year. A second treatment was offered if the IOP rose to baseline. Subsequent TUG treatments typically lasted far longer than the first treatment (beyond the 1 year reported in this study).

In those subjects washed out of their pharmaceutical glaucoma medication, the intraocular pressure measurements after the TUG treatment were equal (within 10%) to or even better (70% of the visits) than their pharmaceutical control for the year of the study.

The subjects tolerated the procedure well with only minimal discomfort noted on the first day post-treatment. There was typically a symptom of only a mild feeling of irritation and a slit lamp finding of mild to moderate conjunctival hyperemia.

A method to treat glaucoma which reduces the issues of pharmaceutical compliance, allergy, and side effects and has the potential for portability could be a significant contribution. It has even more potential for areas of the third world where glaucoma is more prevalent and treatment with medication and/or surgery is unavailable. An updated prototype weighing less than 5 lbs with the same characteristics as that used for this study has now been produced and is in early multicenter clinical trials.

## Conclusion

This is a report of a first-in-human trial of a low-power, low-frequency ultrasound instrument to treat open-angle glaucoma. The findings are supportive of a well-tolerated procedure with a significant decrease in intraocular pressure. Further studies are needed for validation.

## Abbreviations

IOP: intraocular pressure; OD: right eye; OS: left eye; SLT: laser selective laser therapy; TUG: therapeutic ultrasound for glaucoma.

## Competing interests

Dr. Schwartz is the Founder and President of EyeSonix, the company that has developed the TUG treatment. The other authors declare that they have no competing interests.

## Authors’ contributions

DS was responsible for the design of the study, the clinical work necessary, and the initial review and analysis of the data. JS was responsible for the collaboration of the design of the study and review of the manuscript. OK was responsible for the review of the data and substantial help with the analysis of data results. All authors read and approved the final manuscript.
